# Immunogenic Cell Death as a Target for Combination Therapies in Solid Tumors: A Systematic Review Toward a New Paradigm in Immuno-Oncology

**DOI:** 10.7759/cureus.85776

**Published:** 2025-06-11

**Authors:** Imad Barjij, Meryem Meliani

**Affiliations:** 1 Department of Medical Oncology, Faculty of Medicine and Pharmacy of Rabat, National Institute of Oncology, Ibn Sina University Hospital, Mohammed V University, Rabat, MAR; 2 Department of Medical Oncology, Regional Oncology Center of Laâyoune, Regional Hospital Center of Laâyoune, Laâyoune, MAR

**Keywords:** combination therapy, immune checkpoint inhibition, immunogenic cell death, oncolytic virotherapy, photodynamic therapy, radiotherapy, solid tumors

## Abstract

Immunogenic cell death (ICD) represents a distinct form of regulated cell death that triggers robust antitumor immune responses through the release of damage-associated molecular patterns (DAMPs) such as calreticulin (CRT), extracellular adenosine triphosphate (ATP), and high-mobility group box 1 protein (HMGB1). While ICD has emerged as a promising strategy to enhance cancer immunotherapy, its integration into therapeutic regimens remains fragmented. This systematic review aimed to synthesize the experimental evidence on ICD-inducing treatments in solid tumors and assess the convergence of mechanistic pathways and combination strategies. A comprehensive literature search identified 14 eligible studies published between 2010 and 2025, including preclinical in vitro and in vivo investigations of radiotherapy, chemotherapy, photodynamic therapy (PDT), oncolytic virotherapy, and redox- or lysosome-targeted agents. Despite mechanistic diversity, most interventions converged on shared cellular stress responses, notably endoplasmic reticulum (ER) stress and reactive oxygen species (ROS) production, leading to DAMP exposure and dendritic cell maturation. Eight studies incorporated immune checkpoint inhibitors, revealing synergistic antitumor effects and immune memory enhancement. While most studies demonstrated in vivo efficacy, two relied solely on in vitro or ex vivo models. The risk of bias was low in the majority of cases. Collectively, the evidence supports ICD as a central immunologic interface capable of transforming cytotoxic therapies into immune-activating treatments. Future clinical research should prioritize ICD biomarker validation, optimization of treatment timing, and development of personalized ICD-based combination regimens. These findings reinforce the potential of ICD to serve as a unifying framework in the next generation of cancer immunotherapies.

## Introduction and background

Despite the revolutionary impact of immune checkpoint inhibitors (ICIs) in oncology, a large proportion of patients with solid tumors fail to respond due to an immunosuppressive tumor microenvironment (TME) and a lack of spontaneous T-cell infiltration. These *cold* tumors remain a major therapeutic challenge. Among emerging strategies to overcome immune resistance and enhance tumor immunogenicity, immunogenic cell death (ICD) has garnered increasing attention as a mechanism capable of converting a silent tumor cell demise into a coordinated and systemic antitumor immune response [1-3].

ICD is a regulated form of cell death characterized by the release or surface exposure of damage-associated molecular patterns (DAMPs), including calreticulin (CRT), extracellular adenosine triphosphate (ATP), and high-mobility group box 1 protein (HMGB1). These signals act sequentially to facilitate dendritic cell (DC) recruitment and maturation, cross-presentation of tumor antigens, and activation of effector T cells. The result is a transition from local cell death to long-lasting, systemic immune memory, making ICD both a therapeutic endpoint and a platform for combination strategies [4,5].

Originally characterized in response to anthracyclines and conventional radiotherapy, ICD has since been observed with a wide range of interventions, such as photodynamic therapy (PDT), oncolytic viruses, lysosomal-targeting compounds, carbon ion radiotherapy, and nanoparticle-based systems. These approaches engage various pathways, including endoplasmic reticulum (ER) stress, lysosomal lipid peroxidation, reactive oxygen species (ROS) signaling, cyclic GMP-AMP synthase-stimulator of interferon genes (cGAS-STING) activation, or pyroptosis, leading to the exposure of DAMPs and subsequent immune activation. Notably, multiple studies have demonstrated that ICD-inducing therapies can sensitize tumors to ICIs or enhance the efficacy of cancer vaccines [6,7].

Despite this growing preclinical and translational body of research, no prior systematic review has specifically synthesized and analyzed the mechanistic and therapeutic evidence for ICD-inducing strategies in solid tumors. Existing reviews are either narrative, mechanistically focused, or limited to single interventions. Furthermore, ICD biology is complex and context-dependent; the pathways engaged, biomarkers used, and immunological consequences vary considerably depending on tumor type, model, and therapeutic agent. This heterogeneity limits our capacity to identify the most promising ICD-based combination therapies and to establish translational guidelines for biomarker selection and treatment design [8,9].

Our review addresses this gap by systematically evaluating original studies, both experimental and early-phase clinical, that investigate ICD-inducing therapies in solid tumors, published between 2010 and 2025. We selected studies that met rigorous inclusion criteria: use of solid tumor models (murine, human, or organoid), administration of a therapy reported to induce ICD, direct assessment of at least one ICD biomarker (CRT, ATP, HMGB1, heat shock proteins 70 and 90 [HSP70/90]), and (iv) evaluation of immune response or combination benefit with other modalities. Studies focused solely on hematologic malignancies, in silico analyses, or descriptive results without mechanistic or immunological validation were excluded.

Importantly, our review captures a diverse array of therapeutic classes and tumor types, from head and neck squamous cell carcinoma (HNSCC), melanoma, colorectal cancer, and mesothelioma to hepatocellular carcinoma. Out of 1187 screened articles, 14 high-quality studies were ultimately included, spanning preclinical (in vitro and in vivo) and early translational settings, some with clinical correlations. While the majority were experimental, several reported findings with relevance to human samples or ex vivo immune assays, such as vaccine rechallenge, T-cell profiling, or DC maturation. The full details of each study, model used, intervention, mechanistic pathway, ICD markers assessed, combination strategies, immune outcomes, and risk of bias, are documented in a table, which forms the analytical backbone of this review.

To structure this systematic review, we developed a guiding research question using a modified Population-Intervention-Comparison-Outcome (PICO) framework. The population of interest comprised solid tumors studied in both preclinical and clinical settings, including human and murine models. The interventions under investigation were therapeutic strategies known or hypothesized to induce ICD, such as chemotherapy, radiotherapy, oncolytic viruses, or PDT. When applicable, outcomes were compared between monotherapy and combination approaches. The principal outcomes of interest included the expression of canonical ICD biomarkers, activation of antitumor immune responses, evidence of tumor regression, resistance to rechallenge, and improved survival.

This review pursued three specific objectives. First, we aimed to identify and categorize the range of therapeutic modalities capable of inducing bona fide ICD in solid tumors, using both biomarker validation and mechanistic insights. Second, we examined the molecular and cellular pathways involved in ICD induction, with a focus on DAMP release and downstream immune activation. Third, we assessed the potential of ICD-inducing treatments as synergistic partners in combination regimens involving ICIs, cancer vaccines, or other immunomodulatory agents.

By providing a critical synthesis of ICD-inducing interventions and their translational potential, this review aims to inform rational therapeutic design in immuno-oncology and guide future experimental and clinical investigations. Notably, we highlight knowledge gaps, biomarker limitations, and opportunities for combination strategies, all of which are essential for advancing ICD from bench to bedside.

## Review

Methodology

Eligibility Criteria

We defined clear inclusion and exclusion criteria based on the Preferred Reporting Items for Systematic Reviews and Meta-Analyses (PRISMA) 2020 recommendations to ensure methodological rigor, consistency, and relevance. Studies were considered eligible if they met the following conditions: they had to be original investigations, either experimental (including in vitro or in vivo models) or clinical studies such as early-phase trials or case series, that evaluated the capacity of a therapeutic intervention to induce ICD. Eligible models included preclinical murine systems and human or murine-derived cancer cell lines, as well as clinical studies in humans with relevant mechanistic data.

We accepted studies investigating ICD induction through either biological or immunological endpoints. Interventions of interest comprised chemotherapy, radiotherapy, PDT, oncolytic viruses, or any therapeutic modality previously known or hypothesized to induce ICD. To ensure mechanistic validity, all included studies had to evaluate at least one established ICD biomarker, such as CRT surface exposure, ATP release, HMGB1 emission, ER stress, ROS production, or HSP70/90 expression, confirmed via experimental assays.

Only studies published between January 1, 2010, and May 2025 in peer-reviewed and indexed journals (PubMed, Scopus, or Web of Science) were retained. We considered publications in either English or French, although English was preferred due to broader accessibility.

Conversely, we excluded non-original publications such as reviews, commentaries, editorials, and letters, as well as conference abstracts and protocol-only manuscripts. Studies lacking explicit ICD relevance or those that did not assess at least one specific ICD biomarker were also excluded. Purely in silico or bioinformatic studies without biological validation were not eligible. We further excluded articles that focused exclusively on hematologic malignancies or those with incomplete or inaccessible data. Duplicate publications or secondary analyses lacking added value were likewise removed from consideration.

Information Sources

A comprehensive literature search was conducted to identify studies eligible for inclusion. The search covered the period from January 1, 2010, to May 15, 2025, and was performed across three major scientific databases: PubMed (via the NCBI interface), Web of Science Core Collection, and Scopus (Elsevier). In addition to the database search, we manually screened the reference lists of all included articles and relevant review papers to identify potentially relevant studies that may not have been captured by the electronic queries. No limitations were imposed regarding country of origin, source of funding, or institutional affiliation.

Search Strategy

The search strategy was developed using a combination of Medical Subject Headings (MeSH) and free-text keywords related to ICD, DAMPs, and solid tumors. Specific terms included “immunogenic cell death” or “ICD”; “calreticulin” or “CRT”; “ATP release” or “HMGB1”; “DAMPs” or “damage-associated molecular patterns”; and “solid tumor,” “cancer,” or “neoplasm.” In addition, terms referring to therapeutic interventions such as “photodynamic therapy,” “chemotherapy,” “radiotherapy,” and “oncolytic virus” were included. Boolean operators were used to combine search terms effectively. Filters were applied to restrict results to studies published between 2010 and 2025, in either English or French. The search syntax was adjusted according to the requirements of each database platform.

Selection Process

The selection of studies followed a two-step screening protocol based on the PRISMA 2020 guidelines. Two independent reviewers (IB and MM) initially screened the titles and abstracts of all retrieved records (*n* = 1,187). After removing 146 duplicates, a total of 1,041 unique records underwent abstract-level screening. Any disagreements regarding study eligibility were discussed and resolved by consensus between the reviewers. Following this initial screening phase, 65 articles were selected for full-text evaluation.

Among these, 51 articles were excluded for the following reasons: 22 were reviews, commentaries, or editorials; 14 did not assess ICD markers; 7 focused exclusively on non-solid tumors; and 8 consisted of in silico studies or incomplete data. After this selection process, 14 studies met all inclusion criteria and were included in the final synthesis. 

Data Collection Process

Data from each of the 14 included studies were extracted manually using a standardized and predefined table format. This process was carried out independently by two reviewers to ensure consistency and reduce the risk of bias. The following variables were systematically collected for each study: reference and year of publication, study design (clinical or preclinical), model used (human, animal, or cell line), cancer type investigated, ICD-inducing intervention, mechanistic pathways explored, biomarkers assessed, whether combination therapy was applied (yes/no), main tumor or immune outcomes, the assigned level of evidence, and risk of bias.

In cases where discrepancies arose between the two reviewers, a joint review of the original source was undertaken to resolve inconsistencies. No automation tools were used at any stage of the data collection process.

Data Items

For each included study, a series of data items was systematically collected to ensure comprehensive evaluation. The primary outcomes of interest included evidence of ICD induction, specifically characterized by the translocation of CRT, release of ATP, and emission of HMGB1. Mechanistic confirmation of ICD-related pathways, such as ER stress, ROS generation, and pyroptosis, was also documented. Additionally, markers of immune activation were recorded, including DC maturation, T-cell infiltration, and the presence of rechallenge immunity. Tumor regression or control was noted whenever reported.

Secondary outcomes comprised synergy with immune checkpoint blockade or other immunotherapeutic approaches, evidence of abscopal or systemic immune effects, and improvements in survival metrics where available.

Other relevant variables included the type of tumor model (e.g., murine, human, or cell-based), details of any combination strategies employed (including agent identity and administration schedule), and indicators of study quality such as the use of experimental controls, replication procedures, and blinding of outcome assessments.

Risk-of-Bias Assessment

All included studies were subjected to a structured risk-of-bias assessment to ensure methodological transparency and comparability. For studies involving animal experimentation (in vivo), a formal evaluation was performed using the Systematic Review Centre for Laboratory Animal Experimentation (SYRCLE) Risk-of-Bias tool, which is specifically validated for preclinical research in laboratory animals. Each study was independently assessed across the 10 standard domains of the tool, including sequence generation, allocation concealment, baseline comparability, blinding, incomplete outcome data, and selective reporting. Ratings were assigned as “low,” “unclear,” or “high” risk of bias for each domain. Disagreements between reviewers were resolved by consensus discussion. 

For studies that were conducted exclusively in vitro or in ex vivo settings, no validated risk-of-bias tool currently exists. As such, these studies were not assessed using the Systematic Review Centre for Laboratory Animal Experimentation (SYRCLE) framework. Instead, they were appraised based on predefined criteria for methodological rigor, including the use of appropriate experimental controls, biological replicates, and clarity of reporting. Although not a formal bias tool, this appraisal ensured a consistent evaluation across different study types. The inclusion of these studies has been maintained due to their mechanistic relevance, but their interpretive weight was adjusted accordingly.

This dual assessment strategy, combining validated tools with expert judgment, allowed for a rigorous and context-appropriate appraisal of study quality across heterogeneous preclinical designs.

Synthesis Methods

Due to the high degree of heterogeneity in study design, outcome measures, and reporting standards among the included studies, a formal quantitative meta-analysis was not conducted. Instead, a structured narrative synthesis approach was adopted. This synthesis organized the included studies into thematic categories based on several key dimensions: the type of therapeutic intervention used (such as PDT, oncolytic viruses, or chemotherapy), the tumor type or experimental model investigated (e.g., colorectal cancer, melanoma, HNSCC), the specific ICD biomarkers assessed, and whether the intervention was used as monotherapy or in combination with other treatments.

Results

Study Selection

A total of 1,187 records were identified through database searches (PubMed, Web of Science, Scopus). After removing 146 duplicates, 1,041 records were screened by title and abstract. Following the exclusion of 976 non-relevant articles, 65 full-text articles were retrieved for detailed evaluation. Among these, 51 studies were excluded for the following reasons: non-original articles (*n* = 22), lack of ICD biomarker analysis (*n* = 14), non-solid tumor models (*n* = 7), and studies with no biological data (in silico, protocols, or inaccessible texts; *n* = 8).

**Figure 1:**
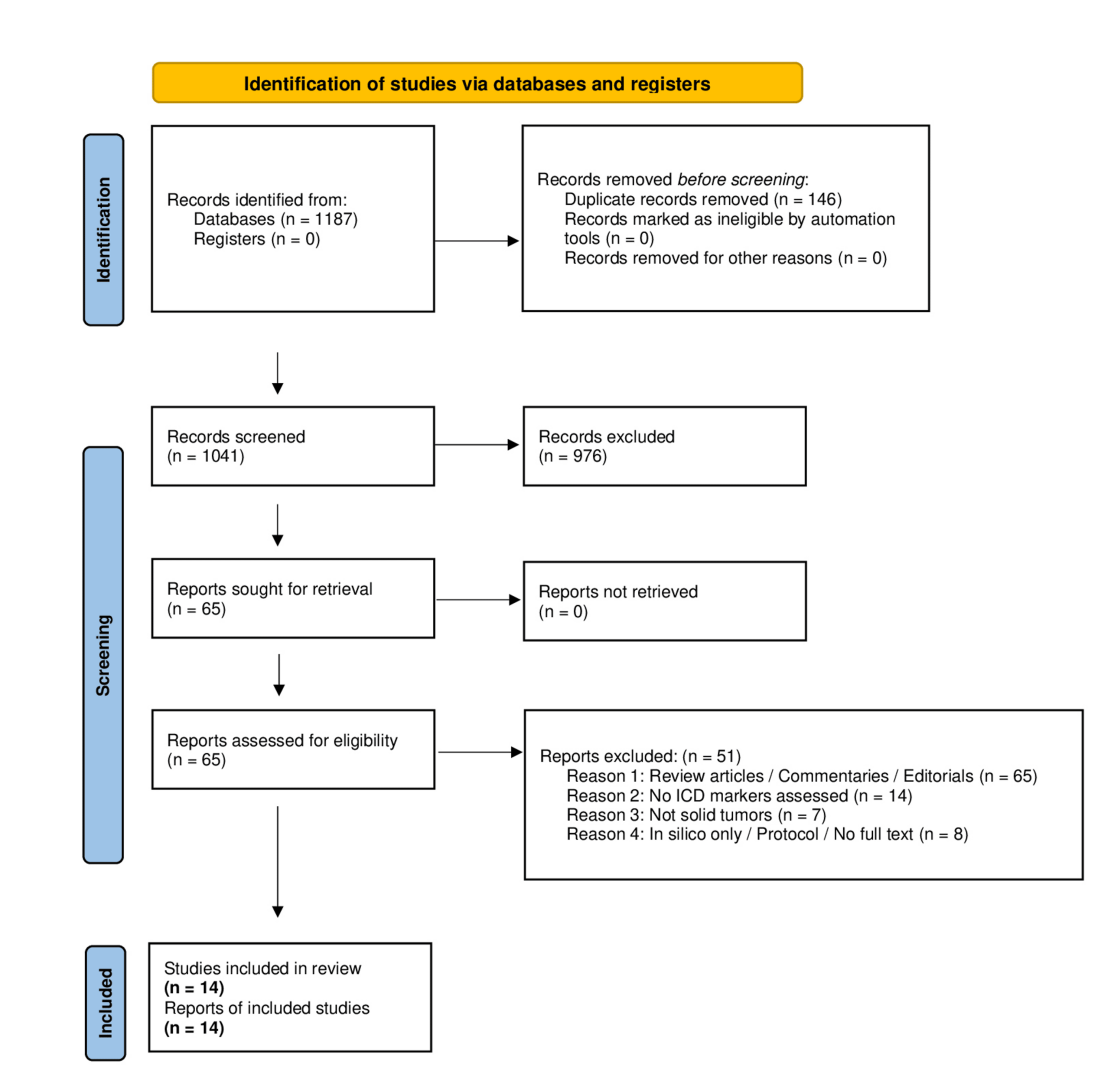
PRISMA 2020 flowchart. Flow diagram illustrating the selection process of studies included in the systematic review, based on PRISMA 2020 guidelines. A total of 1,187 records were identified, 1,041 were screened, and 14 studies met the eligibility criteria and were included in the final synthesis. PRISMA, Preferred Reporting Items for Systematic Reviews and Meta-Analyses

Ultimately, 14 studies met the inclusion criteria and were included in the review. The full selection process is detailed in the PRISMA flow diagram (Figure 1).

Study Characteristics

All 14 included studies were preclinical, except for one study that incorporated primary human tumor samples ex vivo [11]. Most investigations used in vitro and in vivo murine models, with tumor types ranging across melanoma, colorectal, breast, pancreatic, mesothelioma, osteosarcoma, hepatocellular carcinoma, and HNSCC. ICD was induced via radiotherapy, chemotherapy, PDT, oncolytic virotherapy, lysosomal targeting, or nanoparticles. A detailed summary of each study’s characteristics is provided in Table 1.

**Table 1:** Summary of included studies evaluating ICD induction in solid tumors: models, mechanisms, biomarkers, and therapeutic outcomes. ICD, immunogenic cell death; DAMPs, damage-associated molecular patterns; CRT, calreticulin; HMGB1, high-mobility group box 1; ATP, adenosine triphosphate; FACS, fluorescence-activated cell sorting; IF, immunofluorescence; WB, western blot; CLSM, confocal laser scanning microscopy; ELISA, enzyme-linked immunosorbent assay; DC, dendritic cell; MDSC, myeloid-derived suppressor cell; PDT, photodynamic therapy; PPT1, palmitoyl-protein thioesterase 1; LMP, lysosomal membrane permeabilization; LLP, lysosomal lipid peroxidation; TrxR, thioredoxin reductase; NIR-PIT, near-infrared photoimmunotherapy; ROS, reactive oxygen species; GSDME, gasdermin E (involved in pyroptosis); STAT3, signal transducer and activator of transcription 3; CI, combination index; IFN-γ, interferon gamma; IFN-I, type I interferon; IHC, immunohistochemistry; MCS, multicellular spheroids; TS-PDT, talaporfin sodium-based photodynamic therapy; ICB, immune checkpoint blockade

Author/year/reference	Study type	Model used	Cancer type	ICD-inducing treatment	Explored ICD mechanisms	Assessed ICD markers	Combination approach (yes/no)	Main outcomes	Level of evidence	Risk of bias
Mao et al. (2023) [10]	Preclinical (in vitro and in vivo)	Murine SCC7 and 4MOSC2, syngeneic mice (C3H)	HPV-negative Head and Neck Squamous Cell Carcinoma (HNSCC)	Hypofractionated Radiotherapy	DAMPs exposure, adaptive immunity, IFN pathway, PD-L1 induction	CRT (FACS/IF), ATP (luciferase assay), HMGB1 (IF/ELISA)	Yes (anti-PD-L1 ± MDSC depletion)	Tumor regression, protection from rechallenge, enhanced effector T-cell activation, and MDSC-dependent modulation	High (comprehensive in vivo, clinical correlation with human HNSCC samples)	Low (randomized animal studies, proper controls, reproducibility shown)
Rojas et al. (2024) [11]	Preclinical (in vitro and in vivo)	HeLa, MCF-7, 143B; murine syngeneic models (CT26, Renca, B16)	Multiple solid tumors (melanoma, colon, renal, breast)	Novel oncolytic vaccinia virus (IOVA)	ER stress, ICD hallmark induction, fusogenesis, neoantigen-specific immune activation	Surface CALR (FACS), HMGB1 (ELISA), ATP (luminescence)	No adjunct treatment tested, but systemic effect assessed	Strong tumor regression, distal tumor control (abscopal effect), increased survival, and immune memory against rechallenge	High (multi-model, consistent results, in vivo + immune correlates)	Low (randomization, control arms, repeated replicates, strong internal consistency)
Bommareddy et al. (2019) [12]	Preclinical (in vitro and in vivo)	Human melanoma lines (SK-MEL-2/-5/-28, etc.), murine D4M3A	Melanoma	Oncolytic virus (Talimogene laherparepvec, T-VEC)	cGAS-STING signaling, DAMPs release, pro-inflammatory gene signature	CRT (IF), ATP (luminescence), HMGB1 (ELISA)	Yes (anti-PD1 combination tested)	Regression of injected and distal tumors (abscopal effect), activation of CD8+ T cells, and resistance in PD-1-refractory tumors are overcome.	High (multi-model confirmation, mechanism-oriented, in vivo validation)	Low (randomized, well-controlled, sufficient replicates)
Bhardwaj et al. (2023) [13]	Preclinical (in vitro and in vivo)	Human melanoma (A375P, WM35), murine colon (MC38), melanoma (B16F10)	Melanoma, colorectal, and pancreatic	PPT1 inhibitor (DC661)	Lysosomal lipid peroxidation (LLP) → LMP → DAMPs release	CALR (FACS), HMGB1 (ELISA), ATP (luminescence), lipid peroxidation assays	No (monothérapie)	Tumor rejection in MC38 and CT26, T cell activation, and adaptive immunity in immunocompetent mice	High (multiple models, mechanistic confirmation, vaccine assays)	Low (well-controlled, replicates, in vivo validation)
Fucikova et al. (2011) [14]	Preclinical (in vitro, ex vivo with DCs)	Human cancer cell lines (REH, OV90, DU145), primary ovarian tumors	Leukemia, ovarian, prostate	Anthracyclines (doxorubicin, idarubicin)	DAMP exposure: CRT translocation, HSP70/90, HMGB1 release	CRT (FACS), HSP70/90 (FACS), HMGB1 (ELISA)	No (monothérapie)	Induction of tumor-specific T cells, lower Treg induction, enhanced phagocytosis, and DC maturation	Moderate (clinical relevance, but no in vivo efficacy tested)	Moderate (robust flow/FACS data, but lacks in vivo proof)
Deng et al. (2020) [15]	Preclinical (in vitro and in vivo)	Murine (4T1), human breast cancer cell lines (MDA-MB-435, MDA-MB-231, MCF-7)	Breast cancer (murine + human models)	ER-targeted photodynamic therapy (TCPP-TER + NIR)	ROS-induced ER stress → DAMP release	CRT (FACS, WB), HMGB1 (CLSM, WB), ATP (luminescence)	No	Tumor regression, ICD amplification, ER localization essential for response, abscopal-like effect	High (novel mechanism, multiple markers, robust in vivo response)	Low (well-validated model, mechanistic insight, replicates, ER-targeted design)
Jafari et al. (2020) [16]	Preclinical (in vitro + ex vivo DC maturation)	Murine melanoma (B16F10), colon carcinoma (CT26)	Melanoma, colorectal	Doxorubicin ± Stattic (STAT3 inhibitor)	STAT3 inhibition, DAMP release	CRT (FACS), HMGB1 (ELISA), HSP70 (ELISA), IL-12 (ELISA, DC activation)	Yes (DOX+Stattic)	Synergistic ICD enhancement with DOX+Stattic (CI<0.9), DC maturation, IL-12 ↑	Moderate (robust data but no in vivo validation)	Moderate (triplicate, proper controls, ex vivo model only)
Jafari et al. (2022) [17]	Preclinical (in vitro + ex vivo DC maturation)	Murine melanoma (B16F10), colon carcinoma (CT26)	Melanoma, colorectal	Silibinin ± Doxorubicin or Oxaliplatin	DAMPs release, STAT3 inhibition, DC activation	CRT (FACS), HMGB1 (ELISA), HSP70 (ELISA), IL-12 (ELISA, DCs)	Yes (DOX+Silibinin)	Silibinin induces ICD alone and enhances DOX-ICD; synergistic effect with DOX (CI<0.9), IL-12 ↑	Moderate (comprehensive in vitro/ex vivo, no in vivo)	Moderate (repeated assays, but no animal data)
Trempolec et al. (2020) [18]	Preclinical (in vitro and in vivo)	Murine mesothelioma Ab1, Ab12 (BALB/c)	Peritoneal mesothelioma	Photodynamic therapy (OR141) via white LED	ER stress → CRT exposure, HSP90 and HMGB1 release → DC maturation → T-cell priming	CRT (FACS), HMGB1 (WB), HSP90 (WB), IFNγ, perforin	Yes (vs anti-CTLA-4)	Superior tumor control and survival, strong CD8+ and CD4+ activation, long-term immunity	High (Multimodal validation: cell death, DAMPs, DC migration, T-cell effectors, survival; use of appropriate control arms and longitudinal analysis)	Low (Randomization, adequate replicates, multiple markers, well-characterized vaccine protocol)
Wang et al. (2023) [19]	Preclinical (in vitro and in vivo)	Human DLD-1, HCT116; murine CT26 (BALB/c)	Colorectal cancer	Oxaliplatin + DVDMS-derived PDT	ER stress (↑p-eIF2α), DAMP release, ICD marker amplification	CRT (FACS), HMGB1 (ELISA), ATP (ELISA), CD8+ infiltration	Yes	Increased apoptosis, autophagy, enhanced ICD markers, and tumor rejection in the vaccination assay	High (Robust multi-marker ICD confirmation + functional vaccine assay in immunocompetent model)	Low (Reproducible across 3 lines, proper negative/positive controls, validated methods)
Xiao et al. (2021) [20]	Preclinical (in vitro and in vivo)	Murine CT26 (colorectal); CT26 MCSs	Colorectal cancer	MCPP: dual GSH/ROS-responsive nanogel (PTX + P18 PDT)	Pyroptosis via GSDME activation; DAMPs exposure; ROS-mediated mitochondrial stress	CRT (CLSM), HMGB1 (IF), ATP (release assay), LDH, pyroptosis index, CD8+, DC maturation	Yes (ICB + MCPP)	Durable tumor regression, immune memory, enhanced anti-PD1 efficacy	High (Synergistic chemo-PDT, mechanistic depth: pyroptosis, DC maturation, vaccination assay)	Low (Strong in vivo models, multi-omics readouts, immunological relevance)
Xu et al. (2022) [21]	Preclinical (in vitro and in vivo)	HCC cell lines (HepG2, Hepa 1-6); C57BL/6 mice; HCC organoids	Hepatocellular carcinoma	Micheliolide (TrxR inhibitor)	ROS-mediated ER stress → ICD; TrxR inhibition → CRT, ATP, HMGB1 release	CRT (IF), HMGB1 (ELISA), ATP (luminescence), CD4+/CD8+ (flow), DCs maturation	No (monothérapie)	Tumor suppression in organoids + mouse models; memory response in rechallenge assay	High (Novel TrxR target, multiscale models: 2D, organoïde, rechallenge vaccinal)	Low (Well-controlled, mechanistic proof, functional immunoassays, minimal bias)
Yamashita et al. (2023) [22]	Preclinical (in vitro)	Human cancer cell lines (A431, BT-474), murine BALB/3T3	Epidermoid carcinoma, breast cancer	NIR-PIT (Pan-IR700/Tra-IR700) vs PDT (Talaporfin sodium)	Photo-induced ICD (CRT/ATP/HMGB1 release), membrane rupture (necrosis)	CRT (IF), ATP (luciferase), HMGB1 (IF), Annexin/PI, LDH assay	No (comparative mono-approach)	NIR-PIT induced consistent ICD in target cells; TS-PDT required high doses and showed cell line–dependent response	Moderate (Good in vitro depth, but lacks in vivo validation or vaccine assay)	Moderate (Rigorously controlled, multiple assays, but limited to 2D culture)
Zhou et al. (2022) [23]	Preclinical (in vitro and in vivo)	Human U2OS; murine melanoma (B16, S91); C57BL/6 mice	Melanoma, osteosarcoma	Carbon Ion Radiotherapy (CIRT, 5 GyE)	ER stress (↑p-eIF2α), CRT exposure, ATP/HMGB1 release, IFN-I ↑	CRT (FC, IF), ATP (ELISA), HMGB1 (WB, IF), IFN-γ, CD8+ (IHC)	Yes (anti-PD-1)	ICD induction in vitro + in vivo, tumor rejection in vaccine model, survival ↑ with CIRT + anti-PD-1	High (Multimodal in vivo validation, functional immunity, IFN-I, tumor rechallenge)	Low (Robust experimental design, multiple tumor models, repeated biological replicates)

Risk-of-Bias Assessment

Risk of bias was assessed using criteria adapted to experimental oncology. Of the 14 studies, 11 were judged at low risk of bias, mainly due to the use of randomization, biological replicates, control arms, and reproducible outcomes. Three studies were rated as moderate risk, primarily due to the absence of in vivo validation or lack of independent replicates [14,17,22].

The detailed assessment across all domains is summarized in Table 2.

**Table 2:** SYRCLE risk-of-bias assessment for included preclinical in vivo studies. Each study was assessed across 10 domains according to the SYRCLE Risk of Bias tool. “Yes” indicates low risk, “Unclear” indicates insufficient information, and “No” indicates high risk. This assessment was only applied to studies with in vivo animal models. SYRCLE, Systematic Review Centre for Laboratory Animal Experimentation

Study	Sequence generation	Allocation concealment	Baseline characteristics	Random housing	Blinding (performance)	Random outcome assessment	Blinding (detection)	Incomplete outcome data	Selective reporting	Other bias
Mao et al. (2023) [10]	Yes	Unclear	Yes	Unclear	Unclear	Yes	Unclear	Yes	Yes	No
Rojas et al. (2024) [11]	Yes	Unclear	Yes	Unclear	Unclear	Yes	Unclear	Yes	Yes	No
Bommareddy et al. (2019) [12]	Yes	Yes	Yes	Unclear	Yes	Yes	Yes	Yes	Yes	No
Bhardwaj et al. (2023) [13]	Unclear	Unclear	Yes	Unclear	Unclear	Yes	Unclear	Yes	Yes	No
Deng et al. (2020) [15]	Yes	Yes	Yes	Unclear	Unclear	Yes	Unclear	Yes	Yes	No
Trempolec et al. (2020) [18]	Yes	Yes	Yes	Yes	Yes	Yes	Yes	Yes	Yes	No
Wang et al. (2023) [19]	Yes	Yes	Yes	Unclear	Unclear	Yes	Unclear	Yes	Yes	No
Xiao et al. (2021) [20]	Yes	Unclear	Yes	Unclear	Unclear	Yes	Unclear	Yes	Yes	No
Xu et al. (2022) [21]	Unclear	Unclear	Yes	Unclear	Unclear	Yes	Unclear	Yes	Yes	No
Zhou et al. (2022) [23]	Yes	Yes	Yes	Yes	Yes	Yes	Yes	Yes	Yes	No

Overview of ICD-Inducing Strategies

Radiotherapy-based ICD induction: Two studies included in this review investigated the role of ionizing radiation as a trigger for ICD. Mao et al. [10] explored hypofractionated radiotherapy in HNSCC models and reported the induction of robust ICD biomarkers, including CRT exposure, ATP release, and HMGB1 emission. In addition to these hallmarks, adaptive immune responses were observed, notably protection against tumor rechallenge. Furthermore, the therapeutic effect was significantly amplified when radiotherapy was combined with immune checkpoint blockade targeting PD-L1, along with myeloid-derived suppressor cell (MDSC) depletion [10].

Similarly, Zhou et al. [23] assessed carbon ion radiotherapy (CIRT) in preclinical models of melanoma and osteosarcoma. Their findings demonstrated activation of ER stress pathways, particularly through phosphorylation of eIF2α, a key molecular signal associated with ICD. When combined with anti-PD-1 therapy, this approach led to substantial tumor rejection, reinforcing the capacity of radiotherapy not only to induce ICD but also to serve as a potent sensitizer to immune checkpoint inhibitors [23].

Together, these studies highlight the emerging role of radiotherapy as a dual-function modality, capable of both direct cytotoxicity and immunogenic reprogramming, positioning it as a valuable component in combinatorial cancer immunotherapy strategies.

Oncolytic virotherapy: Three studies included in this review explored the immunogenic potential of oncolytic virotherapy. Rojas et al. [11] introduced a novel oncolytic vaccinia virus (IOVA), which demonstrated the ability to induce systemic ICD and establish durable immune memory across several tumor types, including melanoma, colorectal, and breast cancers. This therapeutic effect was achieved without the need for combination with other treatments, highlighting the intrinsic immunostimulatory capacity of IOVA. Observed outcomes included the release of DAMPs, tumor regression, and long-term immune protection [11].

Similarly, Bommareddy et al. [12] investigated the oncolytic herpes simplex virus T-VEC, which is engineered to express granulocyte-macrophage colony-stimulating factor (GM-CSF). Their study revealed that T-VEC activates the cGAS-STING pathway and effectively overcomes resistance to anti-PD-1 therapy in murine melanoma models. These findings reinforce the ability of oncolytic viruses not only to induce ICD but also to reshape the tumor immune microenvironment, thereby enhancing the efficacy of immune checkpoint blockade [12].

Collectively, these results support the use of oncolytic virotherapy as a promising approach for inducing ICD, with strong potential for synergistic effects and abscopal immune responses.

PDT: It emerged as another effective modality for ICD induction, as demonstrated by four independent studies. Deng et al. [15] applied an endoplasmic reticulum-targeted PDT using the photosensitizer TCPP-TER in combination with near-infrared (NIR) light, leading to significant DAMP release, enhancement of ICD signatures, and development of immune memory in breast cancer models [15].

Trempolec et al. [18] utilized a different photosensitizer, OR141, delivered via white light exposure, and showed that this PDT approach could induce long-lasting antitumor immunity in mesothelioma. Their results indicated that the treatment facilitated dendritic cell maturation and T-cell priming, effects that were further amplified by combination with anti-CTLA-4 therapy, resulting in improved survival [18].

Wang et al. [19] investigated a dual strategy combining Oxaliplatin with a DVDMS-based photosensitizer. This approach enhanced ER stress signaling and upregulated ICD biomarkers in colorectal tumor models. Notably, tumor rejection was confirmed through vaccination assays, affirming the immunogenicity of the regimen [19].

In another study, Xiao et al. [20] engineered a dual-responsive nanogel platform (MCPP), combining paclitaxel and a photosensitizer responsive to both ROS and glutathione. This platform induced pyroptotic cell death and immune activation in colorectal cancer, ultimately enhancing the antitumor efficacy of anti-PD-1 treatment [20].

Taken together, these studies emphasize the mechanistic richness of PDT in triggering ICD and its promising role as a combination partner in immunotherapy strategies.

Chemotherapy-induced ICD: A subset of studies examined chemotherapy-induced immunogenic cell death, with a focus on anthracyclines and their modulators. Fucikova et al. [14] offered one of the earliest demonstrations of ICD hallmark induction through ex vivo exposure of primary leukemia and ovarian tumor cells to doxorubicin and idarubicin. The investigators reported calreticulin translocation and dendritic cell maturation, indicating the immunogenic potential of anthracyclines. However, the absence of in vivo validation or tumor control data limited the translational scope of their findings [14].

Jafari et al. [16,17] extended this line of investigation by testing doxorubicin-based combinations with STAT3 inhibitors such as Stattic and Silibinin. Their results confirmed an enhancement in ICD biomarkers, including CRT, HMGB1, and IL-12, using murine melanoma and colon carcinoma cell lines in vitro, as well as DC maturation assays ex vivo. Despite demonstrating STAT3 pathway inhibition as a promising ICD adjuvant, these studies lacked animal model validation, thus constraining conclusions on systemic immune activation or tumor regression [16,17].

Finally, Yamashita et al. [22] reported consistent in vitro ICD induction following near-infrared photoimmunotherapy (NIR-PIT) and PDT in epidermoid and breast cancer cell lines. While mechanistic endpoints such as ATP and HMGB1 release were well documented, no in vivo correlates or rechallenge data were available. Collectively, these chemotherapy-related studies underscore the mechanistic feasibility of ICD induction but highlight the need for in vivo confirmation to fully assess therapeutic potential [22].

Lysosomal and redox-targeting approaches: Two studies examined less conventional, but mechanistically compelling, pathways to induce immunogenic cell death. Bhardwaj et al. [13] identified lysosomal lipid peroxidation (LLP) as a novel trigger for ICD. Their study employed DC661, a selective inhibitor of palmitoyl-protein thioesterase 1 (PPT1), which led to lysosomal membrane permeabilization (LMP) and subsequent release of DAMPs in melanoma and colon cancer models. This mechanism was confirmed across multiple murine systems and linked with robust T-cell activation and tumor rejection, underscoring the immunological relevance of lysosomal stress pathways in oncologic immunotherapy [13].

In parallel, Xu et al. [21] explored the redox-modulating agent micheliolide, a thioredoxin reductase (TrxR) inhibitor, in hepatocellular carcinoma. The compound was shown to initiate ER stress and promote ICD through the release of CRT, ATP, and HMGB1. Notably, its efficacy was validated in vivo using both mouse models and patient-derived organoids, and rechallenge experiments confirmed the induction of long-lasting immune memory. These findings support the therapeutic promise of targeting redox homeostasis as a means of eliciting immunogenic tumor cell death [21].

Together, these studies expand the current understanding of ICD beyond canonical pathways by highlighting lysosomal destabilization and redox imbalance as viable and translationally significant mechanisms.

Synthesis Summary

Among the fourteen studies reviewed, twelve included in vivo validation of immunogenic cell death through endpoints such as tumor regression, rechallenge immunity, and enhancement of immune effector functions in the presence of combination treatments. These validations were crucial in substantiating the functional relevance of ICD markers beyond in vitro correlations.

Two studies, those by Fucikova et al. [14] and Yamashita et al. [22], provided important mechanistic insights based solely on in vitro models. While these studies did not include in vivo confirmation, their contributions to the foundational understanding of DAMP release and immune modulation justified their inclusion, though their findings were interpreted with caution in the absence of functional data.

Across all studies, calreticulin exposure, extracellular ATP release, and HMGB1 emission consistently emerged as the most reliable biomarkers of ICD. Eight studies incorporated combination approaches, most frequently involving immune checkpoint inhibitors such as anti-PD-1 or anti-CTLA-4. In these contexts, ICD inducers significantly enhanced therapeutic efficacy, supporting a synergistic interaction between cytotoxic stress and immune modulation.

Risk of bias was judged to be low in eleven of the fourteen studies, based on clear reporting of methodological controls, randomization, and reproducibility. Importantly, all studies presented mechanistic evidence substantiating ICD as a valid and actionable therapeutic target in oncology.

Discussion

Interpretation of the Findings

This systematic review provides a comprehensive synthesis of the current landscape regarding ICD as a therapeutic target in solid tumors. Our findings reaffirm that ICD is not only a mechanistic concept but also a clinically actionable interface between cytotoxicity and antitumor immunity. Across 14 rigorously selected studies, multiple strategies, ranging from radiotherapy and chemotherapy to PDT and oncolytic viruses, have demonstrated robust ICD induction, supported by canonical DAMPs such as CRT, ATP, and HMGB1.

The review highlights a consistent trend: therapies that trigger ER stress, mitochondrial ROS generation, or lysosomal destabilization are the most potent inducers of ICD. Notably, ER-targeted PDT [15,19] and carbon ion radiotherapy [23] were particularly effective in generating systemic immune responses and memory effects. These observations support the paradigm that intracellular stress compartmentalization plays a central role in immunogenicity [15,19,23].

Another crucial insight is the synergistic value of combination therapies. Eight studies incorporated ICIs or targeted immune modulators alongside ICD-inducing treatments. In these settings, pre-exposure to ICD significantly enhanced ICI efficacy, suggesting a priming effect via DAMP-mediated antigenicity and adjuvanticity. This is particularly relevant in “cold” tumors, such as HNSCC, where turning immunologically silent contexts into inflamed microenvironments is a major therapeutic goal [10].

Interestingly, the choice of ICD markers was largely consistent among studies. CRT exposure, extracellular ATP, and HMGB1 release were the most commonly assessed, reflecting their recognition as “hallmark” indicators. However, emerging markers like pyroptosis index [20], lipid peroxidation [13], and IL-12 secretion [16] reflect an ongoing refinement of the ICD signature, expanding beyond the classical triad. This evolution is crucial, especially as context-dependent immunogenicity may necessitate marker customization by tumor type or mechanism [13,16,20].

Mechanistic Convergence and Therapeutic Architecture

Despite the diversity of therapeutic modalities evaluated across the selected studies, a clear pattern of mechanistic convergence emerged. ICD inducers, PDT, oncolytic virotherapy, radiotherapy, or chemotherapy, were found to act through shared intracellular stress responses and immunostimulatory cascades. These pathways are predominantly centered on organelle-specific perturbations that ultimately culminate in the release of DAMPs and the initiation of adaptive immunity. 

A recurring and central feature was endoplasmic reticulum stress, notably characterized by increased phosphorylation of eukaryotic initiation factor 2 alpha (p-eIF2α), which was reported in six of the fourteen studies. This molecular event appeared to be a consistent upstream signal for the translocation of calreticulin and the release of HMGB1, both of which are key hallmarks of ICD.

ROS generation constituted another common axis, especially prominent in studies involving PDT and redox-targeted therapies. For instance, both Xu et al. [21] and Deng et al. [15] demonstrated that oxidative stress within the ER and mitochondria not only enhanced DAMP exposure but also reinforced the immunogenicity of tumor cell death [15,21].

Additional mechanistic insights revealed activation of innate immune sensors such as the stimulator of interferon genes (STING) pathway, as observed in Bommareddy et al. [12], and modulation of immune tolerance pathways via STAT3 inhibition, as shown by Jafari et al. [16]. These molecular adjuvants were strategically positioned to amplify the immunogenic consequences of ICD and potentiate downstream antitumor immunity [12,16].

Moreover, when ICD inducers were used in combination with immune ICIs, such as anti-PD-1 or anti-CTLA-4 antibodies, a marked amplification of therapeutic responses was consistently observed. These included enhanced infiltration of CD8+ T cells, improved immune memory, and complete tumor rejection in rechallenge models, further supporting the notion that ICD serves not only as a cytotoxic endpoint but also as a powerful immunological primer.

**Figure 2:**
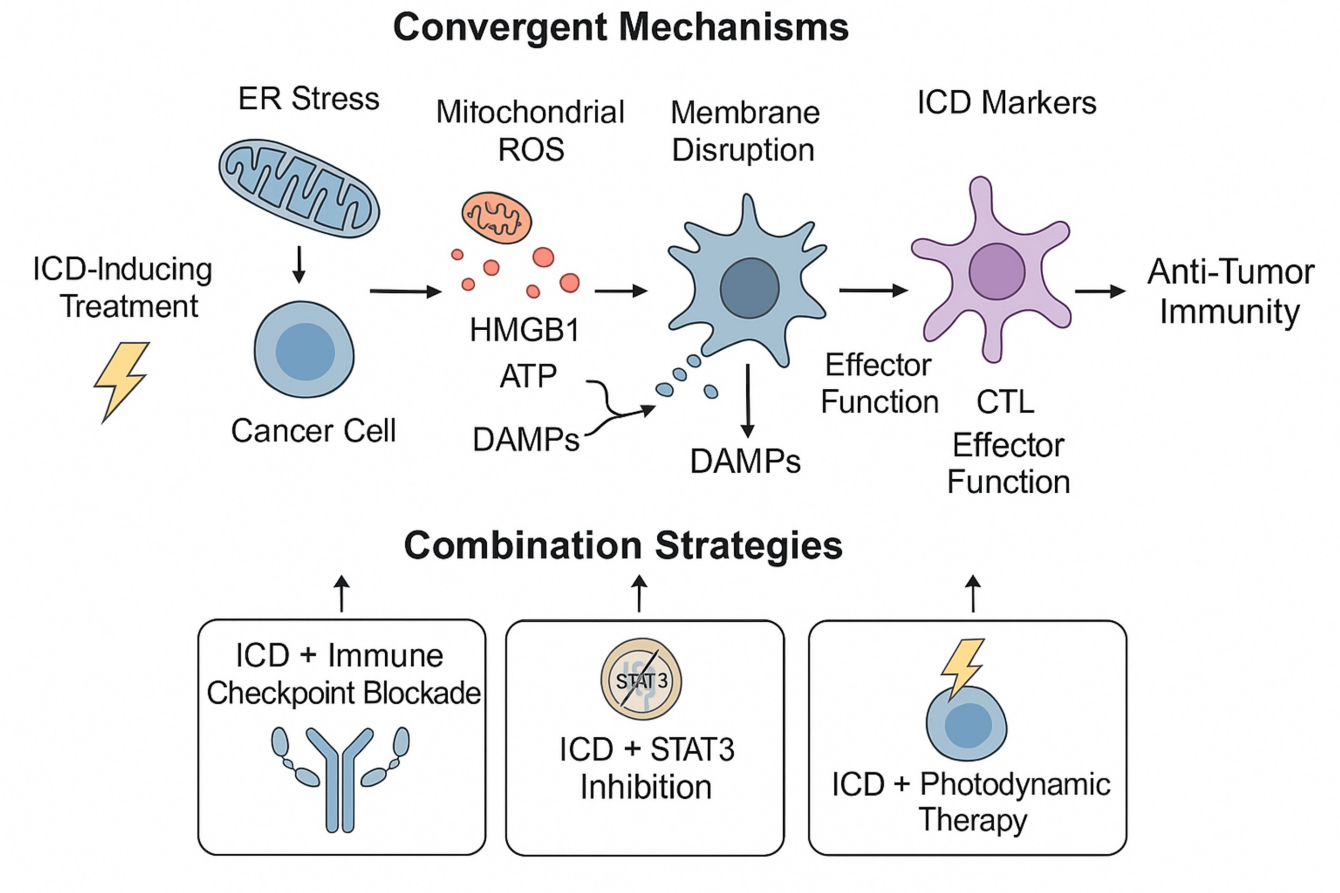
Conceptual model of mechanistic convergence and therapeutic integration for ICD-based strategies in solid tumors. This diagram illustrates how diverse treatment modalities, such as radiotherapy, chemotherapy, PDT, and oncolytic virotherapy, converge on shared cellular stress pathways that promote ICD. Key stress responses include ER stress, ROS generation, lysosomal destabilization, and STING/IFN-I signaling. These trigger the release of DAMPs such as CRT, ATP, and HMGB1, which facilitate dendritic cell maturation and antigen cross-presentation. Combination with immune checkpoint inhibitors (e.g., anti-PD-1, anti-CTLA-4) enhances effector T-cell responses and long-term antitumor immunity. This integrative framework supports the rational design of combination regimens targeting ICD as a central node in cancer immunotherapy. Image created by the authors. No external sources used. No permission required. ICD, immunogenic cell death; PDT, photodynamic therapy; ER, endoplasmic reticulum; ROS, reactive oxygen species; STING, stimulator of interferon genes; IFN-I, type I interferon; DAMPs, damage-associated molecular patterns; CRT, calreticulin; ATP, adenosine triphosphate; HMGB1, high-mobility group box 1; DC, dendritic cell; PD-1, programmed cell death protein 1; CTLA-4, cytotoxic T lymphocyte-associated antigen 4

This conceptual model supports a framework of ICD-oriented combination therapies, where the immunological reprogramming of tumor death becomes a platform for durable tumor control and adaptive memory (Figure 2).

Limitations of the Evidence

Despite the promising trends, several limitations of the included evidence merit discussion. Most studies were preclinical, conducted in murine models or cancer cell lines. Although 12/14 included functional validations in vivo (e.g., tumor regression, rechallenge assays), translational gaps remain regarding dosing schedules, tumor heterogeneity, and immune composition in human cancers [24,25].

In addition, there was some heterogeneity in biomarker evaluation. While CRT, ATP, and HMGB1 were consistently used, quantification methods varied (e.g., FACS vs. immunofluorescence vs. ELISA), which could affect reproducibility. Few studies reported on the kinetics of DAMP exposure, which may be critical to predicting therapeutic timing and synergy. Moreover, very few investigations addressed long-term immune memory or systemic antitumor effects beyond the primary tumor site [26-28].

Finally, only a minority of studies provided side-by-side comparisons of ICD inducers, making it difficult to rank treatments in terms of potency or translational value.

Limitations of the Review Process

While every effort was made to follow rigorous PRISMA 2020 methodology, a few limitations are acknowledged. First, the search was limited to English-language articles, potentially excluding relevant work in other languages. Second, the absence of meta-analysis precludes effect size estimation. This decision was driven by high heterogeneity in study design, outcomes, and models used. Nevertheless, the narrative synthesis was structured to preserve analytical robustness.

Third, while two independent reviewers screened all studies and resolved disagreements by consensus, no automation tools were used, which may limit scalability or generalizability. Furthermore, we did not contact authors to retrieve missing data, which could have clarified ambiguities in methodological detail or outcome reporting.

Limitations of the Included Studies

While this systematic review provides a comprehensive synthesis of ICD-inducing interventions in solid tumors, several limitations must be acknowledged. First, four of the fourteen included studies [14,16,17,22] were conducted exclusively in vitro or in ex vivo settings. These studies, although valuable for their historical or mechanistic insights, such as STAT3 inhibition pathways in Jafari's investigations, lack in vivo validation or rechallenge experiments, which limits the strength of causal inference and translational applicability [14,16,17,22].

Second, the absence of a validated quality assessment tool for in vitro studies led to methodological heterogeneity in evaluating risk of bias. While animal-based studies were assessed using the SYRCLE tool, in vitro studies were appraised based on alternative criteria such as reproducibility, biological replicates, and clarity of reporting. This discrepancy may introduce inconsistency in study-level quality ratings and limit cross-comparisons across preclinical models.

Third, due to substantial heterogeneity in experimental designs, tumor models, and outcome measures, a meta-analysis was not feasible. Instead, a structured narrative synthesis was used, which may be subject to interpretative bias. Furthermore, this review was limited to articles published in English and indexed in three major databases, which may have excluded relevant grey literature or non-indexed studies.

Finally, while our search strategy aimed for breadth and sensitivity, potential publication bias cannot be excluded, especially favoring studies with positive or translational outcomes. Future efforts should prioritize the development of standardized frameworks for evaluating in vitro immuno-oncology research, as well as the integration of multicenter validation studies to enhance generalizability and reproducibility.

Implications for Practice, Policy, and Future Research

The translational relevance of ICD in oncology is increasingly recognized. The findings of this review suggest that ICD-oriented strategies may serve as foundational components of future combination therapies in immuno-oncology. Notably, the integration of ICD into clinical and translational frameworks could provide both mechanistic insight and therapeutic synergy.

From a clinical perspective, personalized treatment design should take into account tumor-specific responsiveness to ICD induction and the kinetics of DAMP exposure. Prospective clinical trials would benefit from the inclusion of ICD biomarkers, not only for response monitoring but also for patient stratification based on immunogenic potential.

The development of ICD-based vaccines, particularly those using ex vivo dying tumor cells, represents a promising avenue. These platforms may complement or enhance the efficacy of existing dendritic cell-based or mRNA vaccine approaches [7,29,30].

At the policy level, there is a strong rationale for incorporating ICD-related endpoints into oncological trial frameworks, such as immunotherapy-adapted RECIST criteria. Doing so may expedite regulatory approval processes for novel combination regimens.

Looking ahead, future research should prioritize several key directions. First, there is an urgent need to validate ICD biomarkers across large, well-annotated patient cohorts to establish their diagnostic and prognostic utility. Second, the exploration of non-classical forms of immunogenic cell death, including pyroptosis, necroptosis, and ferroptosis, should be intensified, using clinically relevant models and endpoints that reflect translational applicability. Third, optimizing the timing and sequencing of ICD inducers in conjunction with immune checkpoint inhibitors and other immunomodulatory agents will be crucial to enhancing therapeutic efficacy. Finally, the integration of systems immunology and single-cell technologies offers an unprecedented opportunity to dissect the molecular and cellular dynamics of DAMP recognition, antigen processing, and adaptive immune priming in real time.

In essence, ICD not only represents a mechanism of tumor cell elimination but also holds the transformative potential to convert dying cancer cells into patient-specific anticancer vaccines. This paradigm shift may fundamentally redefine the landscape of cancer therapy in the coming years.

## Conclusions

ICD represents a pivotal biological phenomenon that bridges tumor cell death with adaptive immune activation. This systematic review consolidates experimental evidence across multiple ICD-inducing strategies, including radiotherapy, photodynamic therapy, virotherapy, chemotherapy, and lysosomal/redox-based agents, applied in solid tumors. While mechanistically diverse, these treatments converge on shared stress pathways such as ER stress, ROS production, and STING signaling, ultimately leading to the release of hallmark DAMPs like calreticulin, HMGB1, and ATP. These signals not only mark tumor cells for elimination but also transform them into effective vaccines by promoting antigen presentation and T cell priming. A key finding of this review is the synergy achieved when ICD inducers are combined with immune checkpoint inhibitors, resulting in enhanced tumor rejection, memory responses, and reversal of immunoresistance in preclinical models. Despite the heterogeneity of study designs and model systems, the convergence of mechanistic and immunological outputs provides a strong rationale for translating ICD into combinatorial cancer therapies. Nevertheless, certain gaps remain. While most studies incorporated in vivo validation, few extended to clinical models, and only a minority explored long-term immunity or systemic responses. Moreover, standardization of ICD markers and functional assays will be essential for future translational success.

In conclusion, targeting ICD offers a promising strategy to amplify the immunogenicity of conventional anticancer therapies. Future research should prioritize the integration of ICD-based biomarkers in clinical trials, optimization of treatment sequences, and development of personalized therapeutic platforms. As cancer therapy continues to evolve, ICD stands as a conceptual and practical cornerstone for designing next-generation immuno-oncology regimens.
